# Age and gender differences in static and dynamic balance of Chinese preschool children

**DOI:** 10.3389/fphys.2022.1013171

**Published:** 2022-10-17

**Authors:** Ruiyuan Li, Meng Liu, Jiefeng Zhu, Ruiqin Li, Huan Zhao, Liqing Zhang

**Affiliations:** ^1^ Sports Coaching College, Beijing Sport University, Beijing, China; ^2^ College of Sports, Xinzhou Teachers University, Xinzhou, China; ^3^ College of Sports, Shanxi Normal University, Taiyuan, China

**Keywords:** static balance, dynamic balance, preschool children, age differences, gender differences

## Abstract

**Objectives:** Balance is a crucial ability of early age, but there is conflicting evidence with regard to age and gender differences in preschool children’s balance ability. Additionally, there are several tools available to measure balance, yet, wide variation in their use has restricted the capacity to synthesize the reference values. Therefore, the primary purpose of this study was to investigate the balance ability of preschool-aged children and determine how it is moderated by age and gender. The analysis pertained to determining whether different testing methods affect the results of static or dynamic balancing ability to provide a basis for normative balance ability data for healthy boys and girls between 3 and 6 years of age.

**Method:** Six hundred and nineteen preschool children (296 boys and 323 girls) aged 3–6 years participated in the study. The static balance (SB) was assessed with children standing on one leg (OST) and in a tandem stance (TS) with respect to time. The balance beam test (BBT) and functional reach test (FRT) were used to evaluate dynamic balance (DB) by measuring the time spent and the distance reached, respectively.

**Result:** The results revealed significant differences in OST with respect to gender (η^2^ = 0.037, *p* < 0.001), TS (η^2^ = 0.026, *p* < 0.001) and FRT (η^2^ = 0.016, *p* = 0.002); the girls performed better than boys on most balance tests except on BBT (η^2^ = 0.000, *p* = 0.596). Age had positive effects on the static and dynamic balance performance on the OST (η^2^ = 0.336, *p* < 0.001), TS (η^2^ = 0.205, *p* < 0.001), BBT (η^2^ = 0.367, *p* < 0.001) and FRT (η^2^ = 0.392, *p* < 0.001). Older children performed better than their younger counterparts. No significant interactions between age groups and sex were found.

**Conclusion:** This study revealed that static and dynamic balance stability in preschool-aged children was affected by gender and age. Gender dimorphism is present in preschool children, older girls displayed better postural stability than boys, and balance performance improved with age. In addition, the study provides age- and gender-specific balance performance reference values for preschool children across multiple methods, which can be used to monitor static and dynamic balance development.

## 1 Introduction

Balance is an essential basic ability of humans, which guarantees an individual’s ability to move. It is a key element that ensures adequate movement capabilities. Children begin learning how to use and integrate the three different sources of sensory information (i.e., visual, vestibular, and proprioception) to maintain balance at 3–6 years of age, with proprioceptive functions maturing by the age of 3–4 years (Steindl et al., 2006), and the structures responsible for motor control are developed by the age of 2–7 years. At the age of 7 years, children’s mechanism of balance adjustment becomes similar to that of the adults (Riach and Hayes, 1987; Assaiante, 1998). Therefore, normal development of the ability to balance in early childhood is a critical part of the developing human balance ability (Shumway-Cook and Woollacott, 1985; Jiang et al., 2018; Giustino et al., 2021). If the development of balance ability is compromised in the early years of life, it is likely to hinder a child’s ability to master complicated movement skills, thus affecting their future ability to participate in sports activities (Mickle et al., 2011; King-Dowling et al., 2020).

Although the development of balance ability is crucial, there is conflicting evidence with respect to age and gender differences in young children’s balance ability. For example, Verbecque et al. (2016) reported that the static balance of young children differed less between 3 and 4 years of age and can only be clearly distinguished with that of 5 year old children as compared to 3 and 4 year old. Jiang et al. (2018) demonstrated that children’s static and dynamic balance indices showed significant differences between the three age groups (3, 4, and 5 years). Similarly, there is conflicting evidence with respect to gender-related differences in balance abilities of the preschoolers. Some scholars have indicated that there is no difference in the static or dynamic balance between boys and girls (Kakebeeke et al., 2012; Latorre-Román et al., 2021). Yet, other studies found that girls have better static and dynamic balance than boys (Deoreo and Wade, 1971; Lee and Lin, 2007; Cadenas-Sanchez et al., 2019; Shams et al., 2020). These findings demonstrate that although balance ability improves with advancing age, yet, age and gender differences in preschoolers’ balance abilities need further exploration.

Furthermore, adoption of field-based methods for measuring balance and postural stability is critical for assessing children in authentic and accessible venues such as schools. Methods such as bipedal stance (BT), tandem stance (TS), one leg hopping (OLH), or one leg stance (OST) for static balance (SB). These methods are commonly employed for measuring children’s SB (Drowatzky and Zuccato, 1967), have high reliability and validity in case of children (Verbecque et al., 2015), and are mostly used for measuring SB of preschool children (eyes open approach in the one leg stance test and the eyes closed approach in the two leg stance test) (Humphriss et al., 2011). Additionally, balance beam test (BBT), functional reach test (FRT), timed up and go test (TUG), four square step test (FSST) used for assessing dynamic balance (DB) can be employed. The BBT and FRT have been commonly used to measure DB in normal children aged 3–6 years (Drowatzky and Zuccato, 1967; Norris et al., 2008; Giacalone and Rarick, 2010; Kasuga et al., 2012a) because of their high reliability and validity (Bartlett and Birmingham, 2003; Gan et al., 2008; Latorre-Román et al., 2021), for a precise evaluation of children’s actual balance abilities displayed in day to day life, rather than their balance performance in laboratory conditions. However, among the limited studies that have examined balance in children, many studies have been restricted to using a laboratory-based force platform to measure balance or postural stability (Steindl et al., 2006). But, laboratory tests are complicated and inconvenient for children, and it is difficult to seek preschool children’s to cooperation in these tests. Hence, the laboratory-based tests are not considered suitable for field studies and large-scale surveys.

Therefore, this study aimed to use more convenient field-based methods and a larger sample to further investigate preschool-aged children’s static and dynamic postural stability, and to determine whether these were moderated by age and gender. It was hypothesized that postural stability improved with age, and children of different ages differed with respect to static and dynamic balance performance. Based on girls employing more mature balance strategies at an earlier age (Kolic et al., 2020), we also assumed that there exist gender differences, with girls being able to balance better than boys.

## 2 Materials and methods

### 2.1 Subjects

The sample size (N = 225) was determined using GPower (version 3.1.9.7; Franz Faul, University of Kiel, Germany) by using α err prob = 0.05; 1-β Err Prob = 0.8; effect size (f) = 0.4; test family = F test, and by analysis of variance (ANOVA) with repeated measures of within-between interaction. Three public kindergartens in Beijing, China, were selected using a convenient whole-group sampling method from which a stratified random sample of 619 preschool children (296 boys and 323 girls; aged 3–6 years), without known pathologies, was randomly selected (Table 1). The physical development of the participants was normal, with no major illnesses, no recent history of trauma that may affect their physical activity, and no physical discomfort such as a cold or fever at the time of testing; they willingly participated in the balance ability test. The 3-year-old group included children between 3 and 3.5 years (3 ≤ *x* < 3.5); the 3.5-year-old group included children between 3.5 and 4 years (3.5 ≤ *×* < 4); the 4-year-old group included children between 4 and 4.5 years (4 ≤ *×* < 4.5); the 4.5-year-old group included children between 4.5 and 5 years (4.5 ≤ *×* < 5); the 5-year-old group included children between 5 and 5.5 years (5 ≤ *×* < 5.5); and the 5.5-year-old group had children between 5.5 and 6 years (5.5 ≤ *×* < 6). Before conducting the test, a detailed explanation was provided to the parents regarding the aims and risks associated with the investigation, with assistance from the kindergarten management, and informed consent was procured from the parents. The study protocol was approved by the Beijing Sport University Institutional Research Commission (Approval number: 2022155H), and the study procedures were carried out in accordance with the Declaration of Helsinki.

**TABLE 1:** Descriptive characteristics of the participants.

Age (years)	n	All		n	Boys		n	Girls	
		Height (cm)	Weight (kg)		Height (cm)	Weight (kg)		Height (cm)	Weight (kg)
3 year old	82	100.10 ± 3.79	15.44 ± 1.70	41	99.85 ± 3.97	15.59 ± 1.85	41	100.35 ± 3.63	15.30 ± 1.55
3 5 year old	102	103.45 ± 3.62	16.32 ± 1.67	45	103.42 ± 3.47	16.55 ± 1.61	57	103.48 ± 3.76	16.14 ± 1.71
4 year old	99	108.01 ± 4.14	17.94 ± 2.07	53	109.38 ± 4.42	18.69 ± 2.24	46	106.43 ± 3.15	17.07 ± 1.43
4 5 year old	130	110.33 ± 4.52	18.78 ± 2.22	63	110.28 ± 4.84	18.70 ± 2.26	67	110.38 ± 4.23	18.86 ± 2.20
5 year old	105	114.05 ± 4.48	19.88 ± 2.39	47	115.02 ± 4.62	20.43 ± 2.08	58	113.26 ± 4.24	19.44 ± 2.55
5.5 year old	101	117.26 ± 4.72	21.25 ± 3.29	47	117.71 ± 4.87	21.60 ± 3.67	54	116.87 ± 4.59	20.94 ± 2.92
Total	619	108.87 ± 4.21	18.27 ± 2.22	296	109.28 ± 4.37	18.59 ± 2.29	323	108.46 ± 3.93	17.96 ± 2.06

### 2.2 Procedures

Before conducting the test, body weight (kg) and height (cm) of the participants were measured without shoes and coats using a balance scale (V-BODY HBF-371, Omron, Japan) and a stadiometer (Ningbo Finer Medical Instruments Co., Limited, Zhejiang, China), respectively. All the tests were conducted in a large kindergarten classroom during morning, and the children were tested in small groups (4 children per group). The order of the four tests was randomized, and they were conducted on the same day. Before participating in the test, the children performed a moderate warm-up exercise that primarily involved jogging and aerobic exercises (Alvarez et al., 2008; Pedersen et al., 2016). Thereafter, the examiner carefully explained the test procedures to the participants, specified the test requirements and demonstrated how to perform the tests ensuring they understood what actually was being tested. After this, the children were familiarized with each test and were allowed two practice attempts on each test. If the child did not understand the task and did not perform the task appropriately, we explained the test methods again and provided a repeat demonstration. Later on, they participated in the formal balance test two to three times. A test taker was responsible for recording the children’s test scores on the test list as soon as the children finished performing.

### 2.3 Balance test

#### 2.3.1 Static balance

##### 2.3.1.1 One-legged stance test

The timed measurement of one-leg standing is used as a static balance measure and has shown good test-retest reliability in typically developing children (Atwater et al., 1990; Humphriss et al., 2011). During the one-leg stand test, the child is asked to stand, for as long as possible, on one leg. The child stands naturally with both eyes looking forward, both arms raised flat at the side in a “T” position, one foot raised, and the support leg straight; the leg is raised and folded backward with the knee joint parallel to the support leg. The stopwatch is started as soon as one foot is lifted and stopped when the child loses balance or touches the floor with the other foot. It is important to note that during the test, when the children lift their leg to lean on or wrap it around the support leg, the arms should not be raised sideways and should not be flat, or the feet should not be raised high enough, and in such cases, they should be re-instructed, and the test should be restarted. The observations were recorded in seconds up to two decimal places. This test was conducted twice, and the longer duration value was used for subsequent calculations. The intraclass correlation coefficient (ICC) for the OST was 0.96.

##### 2.3.1.2 Tandem stance

The Tandem stance is a new item for the component SB. A Tandem stance is a clinical measure of static balance that is considered to assess the postural steadiness with the help of time measurement (Jonsson et al., 2005). The child stands for as long as possible with both arms raised flat on either side and one foot in front of the other, heel-to-toe. Children close their eyes to maintain balance when the command “start” is heard. The stopwatch is started as soon as the child’s eyes closes and is stopped when the child loses equilibrium in the stride or shifts at least one foot out of the original tandem position. The observations were recorded (in seconds), up to two decimal places. The test was executed twice, the longer duration value was used for data analysis. The ICC for the TS was 0.94.

#### 2.3.2 Dynamic balance

##### 2.3.2.1 Balance beam test

The BBT is a test of DB in which the subject walks on a 10 cm wide by 30 cm tall and 3 m long wooden balance beam (Hu et al., 2020; Ke et al., 2021). This test has adequate validity and reliability for testing dynamic balance in children (De Kegel et al., 2010; Latorre-Román et al., 2021). During the balance beam test, children stood on the platform, facing the balance beam with their arms extended straight to their respective sides and raised to their shoulder height. The participants on hearing the word “start,” began to walk forward. The testers started the stop watch at the time the participant started to move and stopped it when the participant’s toes touched the finish line; this is how the time was measured (in seconds), and was recorded up to two decimal places. This test was conducted twice, and the shorter duration value from two tests was considered. The examiner specified that participants could have another try if they fell from the balance beam in the middle of the test, and two attempts were allowed thereafter. The testers arranged for helpers who could protect the participants from falling. The ICC for the BBT was 0.91.

##### 2.3.2.2 Functional reach test

Functional Reach is defined as the maximum distance one can reach forward beyond arm’s length while maintaining a fixed base of support in the standing position. The functional reach test (FRT) was assessed in typically developing children and was found to have high reliability (Volkman et al., 2007; Norris et al., 2008). A piece of masking tape was placed on the floor perpendicular to the wall, and each child was asked to place their toes behind the tape and stand with the side of their left shoulder parallel to the wall. A 1-m graduated straightedge was secured to the wall at the height of the child’s scapula. The FRT was demonstrated and described as follows: “Clench your fist. Raise your arms at shoulder height. Reach forward as far as you can, but don't fall or step forward.” To measure the FR distance, the initial measurement was taken with the child’s arm raised horizontally (approximately 90° of shoulder flexion), using the position of the third metacarpal along the metric ruler. A second measure was taken after reaching, again using the location of the third metacarpal along the metric ruler. This test was performed three times, and the average was taken as the result. The ICC for the FRT was 0.98.

### 2.4 Statistical analysis

Experimental data were processed by IBM SPSS statistical software package (version 26.0, Chicago, IL, United States). All data were presented as “mean ± standard deviation” (M ± SD). A Kolmogorov-Smirnov test (with Lilliefors correction) was used to test the data for normality and Levene’s test for homogeneity of variance. Firstly, two-way ANOVA was applied to the data to determine whether there were any significant (*p* ≤ 0.05) main effects of age or gender or age × gender interactions on the static and dynamic results. When a significant interaction was observed, Tukey’s HSD post-hoc correction was carried to identify the location of the significance. Secondly, one-way ANOVA was used to evaluate differences in the results among the age groups (3, 3.5, 4, 4.5, 5, and 5.5 years) after using a different method. Subsequently, post-hoc pairwise comparisons were performed by employing the Games-Howell approach. *Partial η2* was used to determine the effect size (ES) where the significance was observed, with its strength being interpreted as the following: <0.06 as small, <0.14 as moderate, and ≥0.14 as large (Cohen, 1988). The relative reliability of the test was assessed using the intraclass correlation coefficient of the one-way random-effects model with a single measure (i.e., ICC). The level of significance was set at *p* < 0.05 for all tests.

## 3 Results

In all tests, the numerical value of the child’s index showed a trend of improving year to year, which indicates that the child’s balance ability increased with age. The results showed children in older age groups performing better in than younger age groups, and the older girls are able to balance better than boys. However, there are differences in different indicators.

**FIGURE 1:**
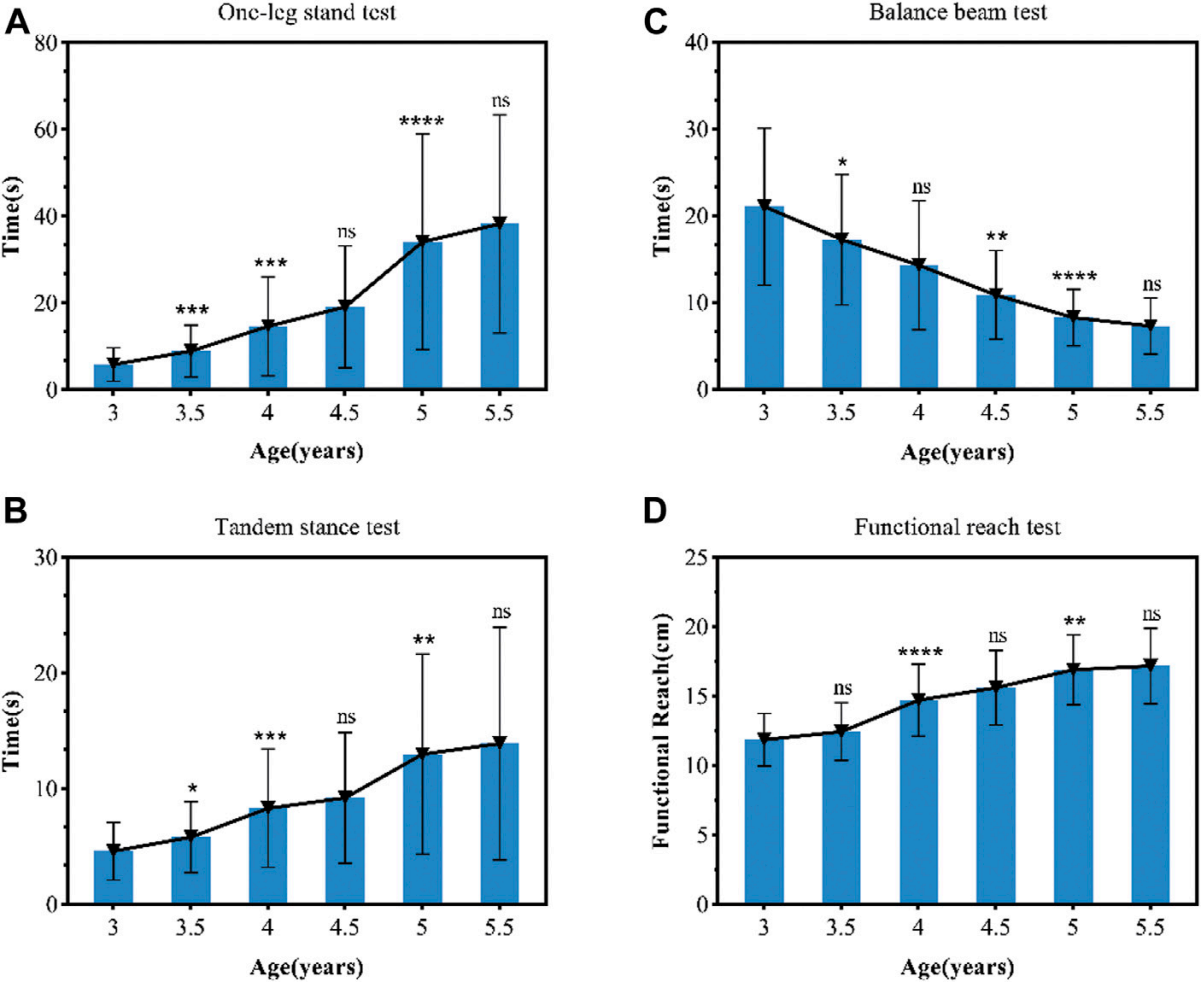
Static and dynamic balance tests result. Static and dynamic balance tests result (mean ± standard deviation) in preschool children from 3 to 5.5 years old group (using one-way ANOVA). Static balance tests of the one-leg stand **(A)** and tandem stance **(B)**; dynamic balance tests of balance beam test **(C)**; and functional reach test **(D)**. The (*) indicates significant difference between the previous age group, *p < 0.05, and ns, no significant difference between the previous age group. In the BBT test, shorter time indicated a better performance.

First, two-way ANOVA models revealed no significant interaction between age and gender on OLS (F = 1.527, *p* = 0.179) and TS (F = 1.365, *p* = 0.236). A significant main effect of age and gender on OLS (age: F = 62.433, *p* < 0.001; gender: F = 23.469, *p* < 0.001) and TS (age: F = 31.326, *p* < 0.01; gender: F = 16.351, *p* < 0.01) was found (refer to Figure 1 and Table 2). No significant interaction effect of age and gender on BBT (F = 0.503, *p* = 0.774) and FRT (F = 0.672, *p* = 0.645), and a significant main effect of age on BBT (F = 70.443, *p* < 0.001) and FRT (F = 78.361, *p* < 0.001). Additionally, significant effects were found with respect to gender on FRT (F = 9.875, *p* = 0.002).

**TABLE 2:** The results of balance performance according to sex and age.

	All	Boys	Girls	*p*-value	η2^p^	3-year-old group	3.5-year-old group	4-year-old group	4.5-year-old group	5-year-old group	5.5-year-old group	*p*-value	η^2^_p_	Age ∗sex	
	(n = 619)	(n = 296)	(n = 323)			(n = 82)	(n = 102)	(n = 99)	(n = 130)	(n = 105)	(n = 101)			*p*-value	*η* ^*2*^_*p*_
One-leg stand(s)	20.56 ± 20.38	16.85 ± 16.59	23.96 ± 22.82^*^	0.000	0.037	5.75 ± 3.86^d^	8.86 ± 5.97^cd^	14.57 ± 11.42^bc^	19.05 ± 14.06^b^	34.06 ± 24.86	38.19 ± 25.12	0.000	0.336	0.179	0.012
Tandem stance(s)	9.29 ± 7.28	8.10 ± 6.14	10.37 ± 8.04^*^	0.000	0.026	4.58 ± 2.49^d^	5.80 ± 3.07^cd^	8.30 ± 5.11^bc^	9.19 ± 5.65^b^	12.97 ± 8.65^a^	13.89 ± 10.05^a^	0.000	0.205	0.236	0.011
BBT(s)	12.78 ± 7.71	12.97 ± 8.23	12.62 ± 7.22	0.596	0.000	21.04 ± 9.05^a^	17.24 ± 7.51^b^	14.28 ± 7.42^c^	10.86 ± 5.11^d^	8.26 ± 3.25^e^	7.29 ± 3.23^e^	0.000	0.367	0.774	0.004
FRT (cm)	14.90 ± 3.14	14.55 ± 3.01	15.23 ± 3.23^*^	0.002	0.016	11.84 ± 1.89^c^	12.43 ± 2.07^c^	14.69 ± 2.59^b^	15.59 ± 2.69^b^	16.89 ± 2.53^a^	17.16 ± 2.71^a^	0.000	0.392	0.645	0.006

^*^ girls vs. boys, *p* < 0.05; a, b, c, d, e is a letter‐marking method used to compare differences between age groups, with identical letters indicating no significant differences; p-values indicate differences between settings using ANOVA, analyses. The data are shown as mean ± SD.

Second, one-way ANOVA models showed that the 3.5, 4, and 5-years old group differed significantly (*p* < 0.001) on OLS and TS. Furthermore, one-way ANOVA models showed that on BBT the 3.5, 4.5 and 5 year old group differed significantly from the 3, 4, and 5 year old group, respectively (*p* < 0.001). On FRT, there was a significant improvement in the functional reach distance of the preschoolers at the age of 4 and 5 years (*p* < 0.001) (see Figure 1).

**FIGURE 2:**
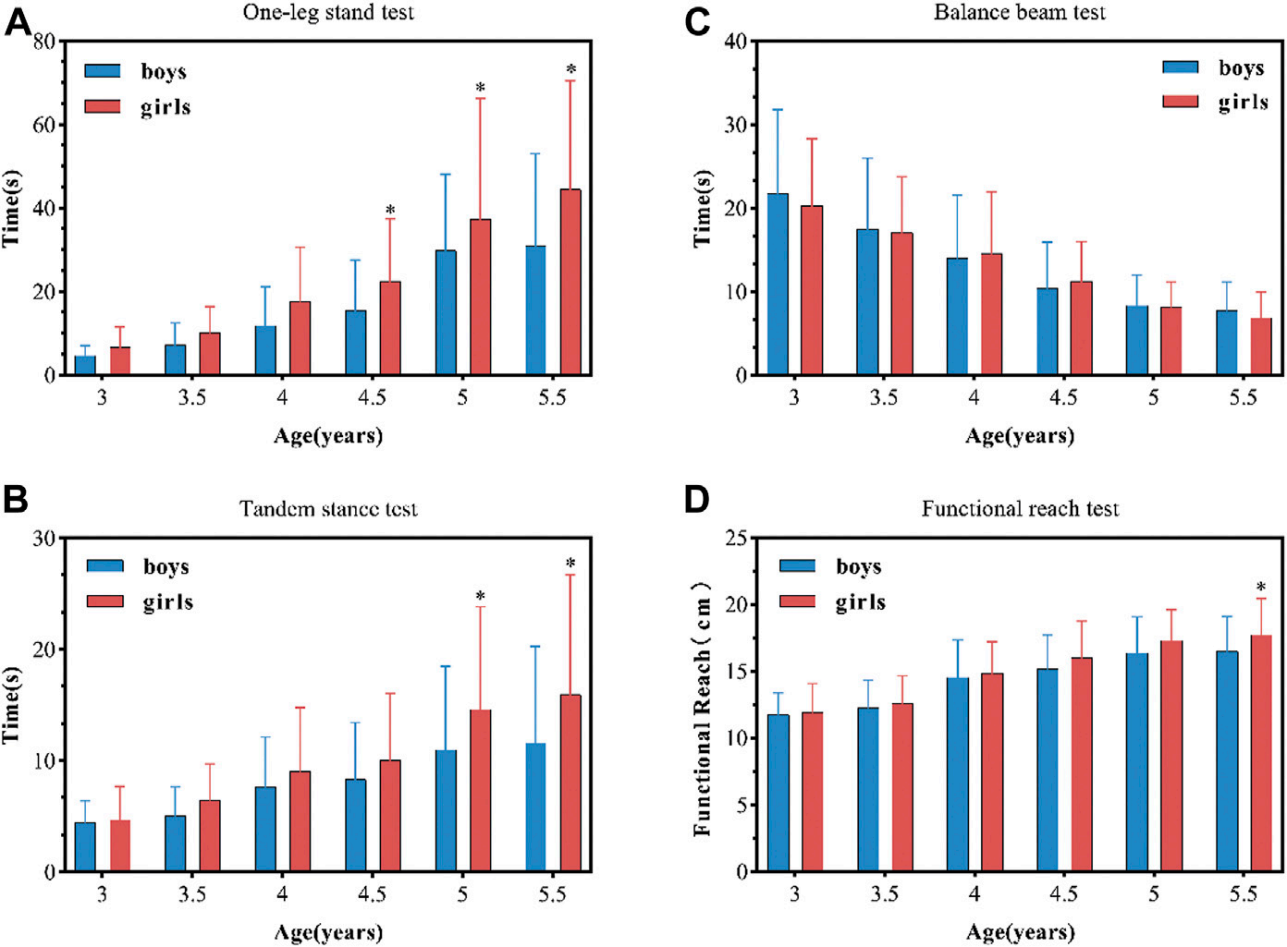
Balance performances of boys and girls. Balance performance (mean ± standard deviation) of boys and girls. The (*) indicates a significant difference between genders at *p* < 0.05. **(A)** One-leg stand test, **(B)** tandem stance test, **(C)** balance beam test, **(D)** functional reach test.

Further comparison of the differences in the balance performance of boys and girls of each age group on the static and dynamic balance ability tests can be found: girls exhibited better performance than boys in most balance tests except for the BBT. Girls performed significantly better than boys in OLS (4.5 years [*p* = 0.018], 5 years [*p* = 0.019], and 5.5 years [*p* < 0.001]), and in the TS (5 years [*p =* 0.005] and 5.5 years [*p* = 0.001]), (see Figure 2). Gender differences in dynamic balance emerged after 5.5 years of age (*p* = 0.014), with girls performing better than boys (see Figure 2).

## 4 Discussion

To the best of our knowledge, this is the first study to report the use of field-based methods to assess the static and the dynamic postural stability simultaneously in preschool children. Our research hypothesis that throughout early childhood, balance develops gradually with age and overall the girls are able to balance better than boys, was confirmed. The findings suggest that both gender and age should be taken into account when measuring balance ability in preschool-aged children. These findings can provide guidance to health, physical education, and school professionals on the use of multiple methods to identify preschool children with high or very low balance and design appropriate movement tasks for boys and girls of different ages.

It has been found that balance control develops progressively throughout early childhood, and the performance of young preschool children is worse on all items of the balance tests as compared to the older children, and this finding is consistent with those of the previous studies (Foudriat et al., 1993; Cumberworth et al., 2007). Since, the ability to integrate sensory information involved in balance control develops between the ages of 3 and 6 years, younger children are less able to suppress inappropriate visual and somatosensory inputs (Forssberg and Nashner, 1982; Cumberworth et al., 2007), and their ability to effectively transition from using somatosensory strategies to using visual strategies is lower as compared to the older children (Woollacott and Shumway-Cook, 1990). Moreover, younger children do not demonstrate integrated postural adjustment, which keeps the pelvis from dropping to the side of the swing leg during foot lift-off (Assaiante et al., 2000). It seems plausible that younger children demonstrate less control over SB and DB associated movements.

These results also showed that children of the age groups (3, 4 and 5 years) differed significantly with respect to the indices of SB and DB, which further supports the hypothesis that balance control improves with maturation (Cumberworth et al., 2007; Shams et al., 2020; García-Liñeira et al., 2021). This is because, as age increases, a recalibration of the sensory processes underlying locomotor balance control occurs (Austad and van der Meer, 2007). The 5-year-old children’s balance performance improved, and they showed significant changes in control strategies as compared to the younger preschoolers (Hao et al., 2021). Additionally, other scholars have demonstrated that by the age of 6–7 years, children achieve postural control like the adults (Shumway-Cook and Woollacott, 1985; Riach and Hayes, 1987; Assaiante, 1998). This finding also confirmed that the age level (3–6 years) is a critical period of rapid development of balance ability in children (Woollacott et al., 1987; Demura et al., 2006). However, in this study, the two indicators of the dynamic balance test reflect different age differences. The children of different age groups 3.5, 4.5, and 5 years differed in their performance on the BBT but did not differ with respect to their performance on the FRT; significant differences in the performance on FRT were observed between the children aged 4 and 5 years, while no significant difference was found in their performance on the BBT (see Figure 2). It is worth noting that results pertaining to development of balance with age may differ on different types of tests (Kasuga et al., 2012b; Butz et al., 2015). Children of different ages have different abilities to understand and complete the balance test tasks, and these abilities are enhanced with the increase of age. As in the study by Verbecque et al. (2016), the number of completions increased with age when young children completed a 40-s postural control task. Data were statistically analyzed when the child was able to perform the task during 40 s, meaning they either passed or failed the condition, and only the data of those who passed were analyzed. This is why a larger number of older children were able to cope with balance tasks, but younger children, when able to perform the task, did similarly to older children. Therefore, we suggest that differences in test methods, conditions, and “how to do statistics” may be responsible for the conflicting evidence with respect to age differences in young children’s balance ability.

The second objective of this study was to analyze whether gender differences existed in preschool children’s balance abilities. Our results indicated that young girls and boys performed similarly well on all tests, with young girls doing slightly better than boys, and the older girls performed better than boys on balance tests except for the BBT, the result being consistent with the findings of other studies that have reported better balance performance in girls as compared to boys of the same age (Kakebeeke et al., 2019; Kolic et al., 2020; Heidt et al., 2021). This may be attributed to several possible factors including earlier maturation of the central nervous structures (Plandowska et al., 2019) that enable sensory integration (Peterson et al., 2006), and the use of more sophisticated postural control strategies (De Bellis et al., 2001). For example, studies of brain maturation have shown that the structure and development of young children’s brain differs between sexes (Lenroot and Giedd, 2006). In girls, the volume of cerebral and gray matter in the frontal and parietal lobes peak earlier than in boys, and the central neural structures also mature earlier (De Bellis et al., 2001). According to Peterson et al. girls are better at using vestibular information and are more capable of integrating their senses (Peterson et al., 2006). Additionally, (Smith et al., 2012), suggested that girls have better postural control in motor conditions where the vestibular system obtains information. Moreover, Chinese girls participate in activities such as dance and gymnastics more often than boys, and these activities are more helpful for the development of balance ability (Jing et al., 2019). This might be attributed to various factors.

The findings of our study also revealed similar results, (i.e., girls performed better than boys in both OST and TS (see Figure 2). Girls are good at skills requiring balance and rhythm and in static activities, such as standing on tiptoe, one-legged balanced stance, rolling, rotating, and other rotary movements, which stimulate the vestibular system (Plandowska et al., 2019). Whereas boys tackle each sensory input related to postural control separately (Peterson et al., 2006), and to a great extent rely on somatosensory feedback (Smith et al., 2012). Shinichi Demural reported that boys performed better than girls in complex dynamic balance tasks (Demura, 1995), such as walking on a balance beam (Venetsanou and Kambas, 2011) which may be because boys perform speed and strength-related activities better than girls (Jing et al., 2019). As a result, girls generally perform better than boys on balance tests, but boys perform better than girls on tests requiring greater muscle strength (Parker et al., 1990; Schedler et al., 2019). Although boys performed well, yet, the performance of participants did not differ largely with respect to gender. In particular, our research also highlighted that boys performed similarly to girls in the BBT task, and the gender difference was not statistically significant, the finding being consistent with that of Demura (1995) and Latorre-Román et al. (2021). It is important to note that most of the previous studies that have considered gender differences in balance in preschool children were conducted on small samples. In studies that include larger samples, the statistical differences in the dynamic balance of preschool children by gender are comparatively small. Gender differences in preschool children’s balance performance need further exploration. Furthermore, the difference in results pertaining to gender differences may be attributed to the different types of test methods, which further suggests that a combination of measures should be used to assess balance in preschool children.

Nevertheless, there were some limitations to the study too. First, this study uses a cross-sectional design for measuring SB and DB in growing children. Future studies can be conducted in the form of longitudinal investigations to observe changes in balance ability with age in the same cohort of children and further confirm the results of this study. Second, the participants in this study were all children from one geographical region. Future studies can be conducted on a larger sample to verify the observations of this study. Moreover, our study did not consider other variables such as participation in extracurricular activities or BMI, and future studies could consider these factors.

## 5 Conclusion

This study aimed to investigate preschool children’s static and dynamic balance ability and to determine whether the balance was moderated by age and gender. We found that both static and dynamic balance stability in preschool-aged children was affected by gender and age. Balance improves with age in the preschool years. Likewise, gender dimorphism as revealed through balance tests is present in preschool children, which increases with age. Overall, girls exhibited better performance than boys in most balance tests. Additionally, our study revealed that the differences in the obtained results may pertain to the use of different methods (i.e., use of OST and TS to evaluate SB, and use of BBT and FRT to evaluate DB). The study provides pertinent insights that are likely to benefit future studies involving large-scale investigations of preschoolers’ balance abilities in real-life everyday situations and help physical education and school professionals employ multiple methods to identify preschoolers’ balance abilities, and accordingly design appropriate movement tasks for boys and girls of different ages.

## Data Availability

The raw data supporting the conclusions of this article will be made available by the authors, without undue reservation.
